# Integrated Metagenomic and Metabolomic Analyses of the Effect of *Astragalus* Polysaccharides on Alleviating High-Fat Diet–Induced Metabolic Disorders

**DOI:** 10.3389/fphar.2020.00833

**Published:** 2020-06-10

**Authors:** Ying Hong, Bingbing Li, Ningning Zheng, Gaosong Wu, Junli Ma, Xin Tao, Linlin Chen, Jing Zhong, Lili Sheng, Houkai Li

**Affiliations:** ^1^ Functional Metabolomic and Gut Microbiome Laboratory, Institute of Interdisciplinary Integrative Medicine Research, Shanghai University of Traditional Chinese Medicine, Shanghai, China; ^2^ Huzhou Key Laboratory of Molecular Medicine, Huzhou Central Hospital, Affiliated Cent Hospital Huzhou University, Huzhou, China

**Keywords:** *Astragalus* polysaccharides, metagenomic, metabolomics, metabolic disorders, hepatic steatosis

## Abstract

Most herbal polysaccharides possess multiple benefits against metabolic disorders, such as non-alcoholic fatty liver disease (NAFLD) and obesity. However, the underlying mechanisms are largely unknown. Here, male C57BL/6J mice were fed with chow or high-fat diet (HFD) with or without *Astragalus* polysaccharides (APS) supplementation, and gut microbial profile and metabolite profile were studied by metagenomic sequencing and untargeted metabolomics, respectively. APS was effective in alleviating HFD-induced metabolic disorders, with the alteration of gut microbiota composition and function. A total of 188 species, which mainly from Bacteroidetes, Actinobacteria, Firmicutes, and Proteobacteria phyla, and 36 metabolites were markedly changed by HFD and revered by APS. Additionally, the altered glutathione metabolism and purine metabolism pathways were identified by both metagenomic function analysis and metabolite pathway enrichment analysis. Furthermore, the gut microbial alteration was associated with the changes of key intestinal metabolites. We found 31 and 20 species were correlated with purine metabolism and glutathione metabolism, respectively. Together, our results showed significant metagenomic and metabolomic changes after HFD feeding and APS intervention, revealed the potential correlation between gut microbial species and metabolites, and highlighted mechanisms of herb-derived polysaccharides by modulating gut microbiome and host metabolism underlying their benefits on metabolic disorders.

## Introduction

Metabolic disorders, such as non-alcoholic fatty liver disease (NAFLD) and obesity, represent hugely problems concerning the health worldwide (Jung and Choi, 2014). Significant interest has recently focused on the effect of gut microbiota in metabolic disorders (Cani, 2019). Gut microbiota is a complex microbial community with highly interactive microorganisms that maintain a close interplay with its host. Emerging evidence has revealed that gut microbiota plays an essential role in prevention and treatment of human diseases, such as obesity and NAFLD (Boulange et al., 2016). Changes in the diversity and composition of gut microbiota directly affect host physiology (Bennett et al., 2013; Yoshimoto et al., 2013; Khan et al., 2016). In addition, the metabolic potential of gut microbiota has been identified as a contributing factor to health (Dahiya et al., 2017). Gut dysbiosis and subsequently altered metabolite profile can lead to many health issues. Although the underlying mechanisms require further investigation, there is an increasing body of evidence that the bacterial metabolites, like short chain fatty acids (SCFAs), bile acids, and tryptophan metabolites, are important modulators of host physiology (Cani, 2019). Therefore, gut microbiota and its metabolites have a pivotal role in the maintenance of physiologic and metabolic homeostasis of the host. Targeting gut microbiota and its metabolites might be a potential therapy for the treatment and prevention of metabolic disorders.

Plant polysaccharides are natural macromolecules that are widely present in various herbs with medicinal properties such as anti-inflammation, anti-virus, and immune modulation (Mao et al., 2007; Gu et al., 2015), as well as metabolic benefits (Chang et al., 2015). *Astragalus* polysaccharides (APS) are extracted from *Astragalus mongholicus* Bunge, a frequently used herbal medicine with established efficacy in lowering plasma lipids, improving insulin sensitivity (Zou et al., 2009; Ke et al., 2017), and ameliorating metabolic risk in metabolically stressed transgenic mice (Huang et al., 2017). Given the non-absorptive properties of polysaccharides in the gastrointestinal tract, the metabolic benefits of polysaccharides are usually associated with modulation or recovery of gut dysbiosis such as the anti-obesity effects of the polysaccharides extracted from *Ganoderma lucidum* and *Hirsutella sinensis* (Chang et al., 2015; Wu et al., 2018). However, the effect of plant polysaccharides on host metabolism is still largely unknown.

In the present study, we proved the effect of APS in attenuating metabolic disorders in high-fat diet (HFD)-fed mice. Cecum metabolomics and bacteria composition was analyzed by liquid chromatography/mass spectrometry (LC/MS)-based untargeted metabolomics and metagenomic sequencing, respectively. Our results showed APS was effective in reversing HFD-induced changes of gut microbial structure and function as well as gut metabolites. Additionally, through both metagenomic function analysis and metabolites pathway enrichment analysis, we filtrated out two metabolism pathways, purine metabolism pathway and glutathione metabolism pathway, which might be important for hepatic steatosis lowering effects of APS. Together, the present study provided new evidences for the beneficial effect of APS on regulating metabolic disorders at both metagenomic and metabolomic levels.

## Materials and Methods

### Preparation of APS Extracts

APS was provided by Ci Yuan Biotechnology Co., Ltd. (Lot# 20140504, Shanxi, China) with 90% purity of polysaccharides from *Astragalus mongholicus* Bunge. Briefly, polysaccharides were extracted from *Astragalus mongholicus* Bunge with distilled boiling water, and the supernatant was condensed and precipitated with 70% ethanol. Crude polysaccharides extract experienced deproteinization by sevage method before dialysis, which was then lyophilized for subsequent monosaccharide analysis and experiment (Li et al., 2003).

### Characterization of Monosaccharide Composition of APS

The extracted APS was hydrolyzed into monosaccharides with trifluoroacetic acid, and the hydrolyzed monosaccharides from APS and monosaccharide standards were acetylated according to a previous method (Lin et al., 2016). The acetylated samples were then analyzed by Agilent Technologies 7890B gas chromatograph (GC, USA) equipped with 3% OV-225/AW-DMCS-Chromosorb W column (3 mm × 2.5 m). The heating program for the GC analysis was as follows: the initial temperature was 140°C, and increased to 198°C at a rate of 2°C/min and maintained for 4 min, then the temperature was increased to 214°C with a temperature gradient of 4°C/min, and then increased to 217°C at the speed of 1°C/min and kept 4 min. Finally, the temperature increased to 250°C at the rate of 3°C/min and held constant for 5 min. The APS used in our current study was composed of five monosaccharides including rhamnose (1.6%), arabinose (23.39%), xylose (0.84%), glucose (70.55%), and galactose (3.61%).

### Animal Study

After 1-week accommodation, 15 mice were treated with chow diet (Con, 16.5% calories from fat, SHOOBREE), HFD (60% calories from fat, Research Diet, D12492) with or without APS for 14 weeks, respectively. Since our preliminary data showed 8% APS supplemented in diet had the most significantly metabolic protective effect than 2% and 4% APS, the finial concentration of 8% APS was used in this study. The experiments were conducted under the Guidelines for Animal Experiment of Shanghai University of Traditional Chinese Medicine and the protocol was approved by the institutional Animal Ethics Committee. At the end of the experiment, mice were sacrificed after anesthesia with 1% pentobarbital sodium solution intraperitoneally. Serum, cecum contents, and tissue samples were collected, weighted, and immediately frozen in liquid nitrogen and stored at −80°C for further analysis.

### Histological Evaluation on the Degree of Hepatic Steatosis

Liver tissues were fixed with 10% neutral formalin for 24 h, embedded in paraffin and stained with hematoxylin-eosin staining (H&E) using a standard protocol. The degree of hepatic steatosis was evaluated according to previous publication in a blinded way (Peng et al., 2009). The criteria for scoring: grade 0, no hepatocytes involved; grade 1, 1–25% of the hepatocytes involved; grade 2, 26–50% of hepatocytes involved; grade 3, 51–75% of hepatocytes involved; and grade 4, 76–100% of hepatocytes involved.

### Metagenomics

About 100 mg of cecum content were used for bacteria DNA extraction using a fecal DNA extraction kit. Bacterial DNA samples were fragmented to an average size of about 300 bp using Covaris M220 (Gene Company Limited, China) for paired-end library construction using TruSeqTM DNA Sample Prep Kit (Illumina, San Diego, CA, USA). Adapters containing the full complement of sequencing primer hybridization sites were ligated to the blunt-end of fragments. Paired-end sequencing was performed on Illumina HiSeq4000 platform (Illumina Inc., San Diego, CA, USA) at Majorbio Bio-Pharm Technology Co., Ltd. (Shanghai, China) using HiSeq 3000/4000 PE Cluster Kit and HiSeq 3000/4000 SBS Kit according to the manufacturer's instructions (www.illumina.com). Sequence data associated with this project have been deposited in the NCBI Short Read Archive database (Accession Number: PRJNA615253). Adapter sequence were stripped from the 3' and 5' end of paired end Illumina reads using SeqPrep (https://github.com/jstjohn/SeqPrep). Low-quality reads (length < 50 bp or with a quality value < 20 or having N bases) were removed by Sickle (https://github.com/najoshi/sickle). Reads were aligned to the Mus musculus genome by BWA (http://bio-bwa.sourceforge.net) and any hit associated with the reads and their mated reads were removed. Data were assembled using MEGAHIT (https://github.com/voutcn/megahit) (Li et al., 2015), which makes use of succinct de Bruijn graphs. Contigs with the length being or over 300 bp were selected as the final assembling result, and then the contigs were used for further gene prediction and annotation. Open reading frames from each assembled contig were predicted using MetaGene (http://metagene.cb.k.u-tokyo.ac.jp/) (Noguchi et al., 2006). The predicted open reading frames with length being or over 100 bp were retrieved and translated into amino acid sequences using the NCBI translation table (http://www.ncbi.nlm.nih.gov/Taxonomy/taxonomyhome.html/index.cgi?chapter=tgencodes#SG1). All predicted genes with a 95% sequence identity (90% coverage) were clustered using CD-HIT (http://www.bioinformatics.org/cd-hit/) (Fu et al., 2012), the longest sequences from each cluster were selected as representative sequences to construct non-redundant gene catalog. Reads after quality control were mapped to the representative sequences with 95% identity using SOAPaligner (http://soap.genomics.org.cn/) (Li et al., 2008), and gene abundance in each sample were evaluated. Representative sequences of non-redundant gene catalog were aligned to NCBI NR database with e-value cutoff of 1e-5 using BLASTP (Version 2.2.28+, http://blast.ncbi.nlm.nih.gov/Blast.cgi) for taxonomic annotations.

### Sample Preparation for Metabolomics Study

For the metabolomics analysis, 10-mg cecum content was added to 200-µl water and homogenized. The homogenate was added to 800 µl of ACN: MeOH (1:1, v/v), vortexed for 30 s, and sonicated for 10 min. After overnight incubation at −20°C, samples were centrifuged at 12,000 g for 15 min at 4°C. The supernatant was transferred into a clean dry tube and dried with nitrogen at 30°C, the residue was reconstituted with 100 µl of ACN: H_2_O (1:1, v/v), vortexed for 30 s, sonicated for 5 min in an ice bath, then centrifuged at 12,000 g for 15 min at 4°C.

### HPLC-QTOF/MS Analysis

Chromatographic analysis was performed using a Shimadzu HPLC system (Nexera XR LC-20AD, Japan) equipped with an ACQUITY UPLC BEH C18 column (2.1 × 100 mm, 1.8 µm). The mobile phase A consists of 0.1% formic acid in H_2_O, mobile phase B was ACN. The gradient was used as follows: 1% B, 0–1.5 min; 1%~99% B, 1.5–13 min; 99% B, 13–16.5 min; 99%~1% B, 16.5–16.6 min; 1% B, 16.6–20 min. The column temperature was 30°C, flow rate was 0.3 ml/min, and the volume of injection was 2 µl for each run.

The metabolomics profiling analysis was performed on an SCIEX Triple TOF 5,600+ with information dependent acquisition (IDA). For the positive mode, the collision energy (CE) spread were set as 40 and 10 eV, declustering potential (DP) set at 60 V, the ion spray voltage floating (ISVF) set at 5,500 V, and the temperature set to 550°C. For the negative mode, the CE spread were set as −40 and −10 eV, DP set at −60 V, the ion ISVF set at 4,500 V, and the temperature set at 450°C. The other source same parameters settings in the two modes were as follows: the ion source gas1 and gas2 were set at 60 psi with curtain gas was set at 35 psi, the TOF/MS full scan was operated with the mass range was 60–1,000 Da and the TOF-MS/MS full scan was operated with the mass range was 25–1,000 Da, and the accumulation time was 0.15 s. The mass spectrometer was automatically calibrated by the calibration delivery system (CDS) once every six injections.

### Metabolomics Data Processing and Metabolites Identification

The wiff data were imported to the Progenesis QI (Waters, Milford, MA, USA) for data processing. The converted files were calculated for generation of alignment, peak picking, deconvolution, filter data and identifying compounds. For the identification of potential biomarkers, several online databases, such as the HMDB (http://www.hmdb.ca/) and LIPIDMAPS (http://www.lipidmaps.org/) were selected for metabolite identification based on exact mass measurement (mass error < 10 ppm) obtained from HPLC-QTOF/MS. Other parameter settings were designed as default for data processing automatically. A data matrix containing retention times, accurate masses, and peak intensities was exported into SIMCA-P 13.0 software (Umetrics, Umeå, Sweden) for principal component analysis (PCA).

### Statistical Analysis

Data are shown as means ± sem unless otherwise noted. Multiple comparisons were performed by using one-way ANOVA followed by Tukey's honest significant difference *post hoc* test with SPSS software (21.0). *p* < 0.05 was considered statistically significant.

## Results

### APS Attenuates HFD-Induced Metabolic Disorders

**Figure 1:**
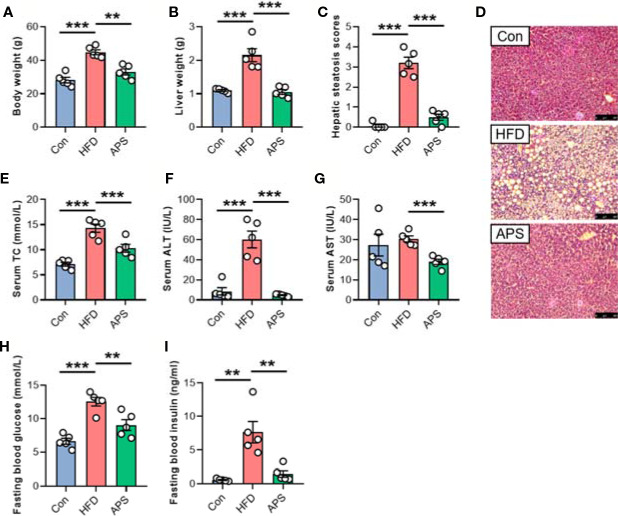
APS attenuates hepatic steatosis in HFD-fed mice. Male C57BL/6J mice (4-week-old) were treated with chow-diet (Con) or high-fat diet (HFD) with or without APS supplementation (8% APS in HFD) for 14 weeks. **(A)** Body weight. **(B)** Liver weight. **(C)** Hepatic steatosis scores. **(D)** Representative photomicrographs of liver tissue with H&E staining (magnification, ×200, 50 μm). **(E)** Serum total cholesterol (TC) level. **(F)** Serum alanine transaminase (ALT) level. **(G)** Serum aspartate aminotransferase (AST) level. **(H)** Fasting blood glucose level. **(I)** Fasting blood insulin level. *n* = 5, **p < 0.01, ***p < 0.001.

To explore the effect of APS on improving metabolic disorders, 4-week-old mice were fed with chow diet, or HFD with or without APS supplementation for 14 weeks. Administration of HFD resulted in significant increases of body weight, liver weight, and hepatic steatosis as revealed by H&E, while APS supplementation reversed these changes (**Figures 1A–D**). In addition, APS significantly reduced serum total cholesterol (TC), alanine aminotransferase (ALT), aspartate aminotransferase (AST), fasting blood glucose, and insulin levels, which were increased by HFD (**Figure 1E-I**). These results indicated that APS was effective in attenuating HFD-induced metabolic disorders in mice.

### APS Reverses Gut Dysbiosis in HFD-Fed Mice

**Figure 2:**
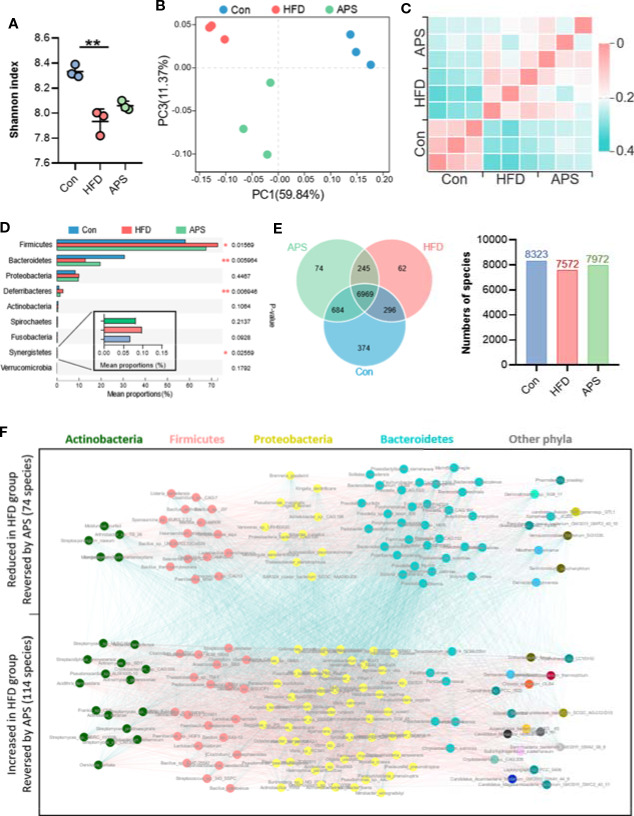
APS reverses gut dysbiosis in HFD-fed mice. Cecum samples of Con, HFD, and APS groups were analyzed with metagenomics. **(A)** Shannon index. **(B)** Bray_curtis based PCoA analysis followed by Permutational Multivariate Analysis Of Variance (PERMANOVA, *R^2^*: 0.695, *p-value*: 0.005, *p.adjust*: 0.005). **(C)** Bray_curtis based distance matrix. **(D)** Multigroup difference analysis of the top 9 abundant phyla. **(E)** Venn diagram illustrating the overlap of species in intestinal microbiota among the samples and number of species in three groups. **(F)** Co-occurrence network deduced from 188 differential species significant changed in HFD group compared to Con group and restored in APS group. Red edges, Spearman's rank correlation coefficient > 0.8, *p* < 0.01; blue edges, Spearman's rank correlation coefficient < −0.8, *p* < 0.01. *n* = 3 per group. **p < 0.01.

Since most plant-derived polysaccharides are non-absorbable, we hypothesized that the effect of APS was probably associated with the modulation of gut microbiota. Cecum contents were collected and used for metagenomics sequencing. An average of 92.8 ± 1.5 (SEM) million reads per sample were generated. Shannon index was reduced significantly in the HFD group and increased by APS supplementation, indicating APS was effective in increasing microflora diversity (**Figure 2A**). Bray-Curtis PCoA showed clear separation among groups, which was consistent with the heatmap of Hierarchical clustering (**Figures 2B, C**). In addition, taxonomic profiling of top 9 most abundance phylum indicated that HFD obviously increased the relative abundance of Firmicutes, Deferribacteres, and Synergistetes phyla, and reduced the relative abundance of Bacteroidetes phylum, whereas APS reversed above changes (**Figure 2D**).

In order to find the specific bacterial species which might mediate the metabolic benefits of APS, we analyzed metagenomics data at species level. Total 8,323 species were annotated in Con group, while only 7,572 species were annotated in HFD-fed mice. Different with the reduced species number found in HFD group, APS supplement increased the species number to 7,972 (**Figure 2E**). In addition, a total of 188 species which differentially changed by HFD and reversed by APS supplement were determined with the double criteria of both fold change ≥ 2 (or ≤ 0.5) and *p* < 0.01. The differential species were mainly from Actinobacteria, Firmicutes, Proteobacteria, and Bacteroidetes phyla. Among them, 74 species were reduced in HFD group and reversed by APS supplement which mostly from Bacteroidetes phylum, while the other 114 species showed opposite changes which mostly from Actinobacteria, Firmicutes, and Proteobacteria phyla (**Figure 2F**). These data suggested APS played an essential role in regulating gut microbiota composition. APS supplementation was effective in reversing HFD induced dysbiosis, which might be associated with the improved metabolic disorders.

### APS Improves Metabolic Function of Microbiome in HFD-Fed Mice

**Figure 3:**
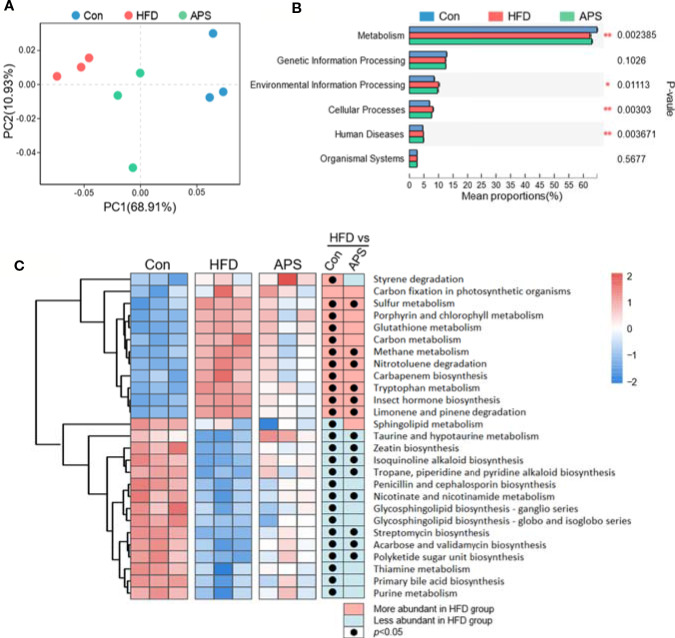
APS regulates gut microbial function in HFD-fed mice. **(A)** Bray_curtis based PCoA analysis in KEGG orthologys (KOs) level followed by Permutational Multivariate Analysis Of Variance (PERMANOVA, *R^2^*: 0.745, *p-value*: 0.002, *p.adjust*: 0.002). **(B)** Multigroup difference analysis in KEGG pathway at level 1. *p < 0.05, **p < 0.01. **(C)** LDA Effect Size (LEfSe) Analysis of gut microbial function at level 3 of metabolism was profiled among three groups. Heatmap of the relative abundances of the metabolic pathways with the criteria of LDA > 2. The black dots mean significant difference (*p* < 0.05) between HFD and Con groups or between HFD and APS groups.

The changes of gut microbiota structure are always accompanied with the alternation of gut microbial function. Hence, we further investigated the functional consequences after APS supplementation. Bray-Curtis PCoA based on Kyoto Encyclopedia of Genes and Genomes (KEGG) orthologs level (KOs) showed clear separation among groups, with APS group clustered between Con and HFD groups (**Figure 3A**). At KEGG level 1 level, the top differential altered pathways were metabolism, environmental information processing, cellular processes, and human diseases. Among them, the metabolic pathway had the highest proportion (**Figure 3B**). Then, we further studied the KEGG level 3 pathway under metabolism. By LDA Effect Size (LEfSe) Analysis, 27 metabolic pathways were screened with the criteria of LDA > 2 (**Figure 3C**). Among them, six metabolic pathways were increased by HFD and reversed by APS supplement significantly, including tryptophan metabolism, methane metabolism, sulfur metabolism, insect hormone biosynthesis, limonene and pinene degradation, and nitrotoluene degradation. Meanwhile, eight metabolic pathways were reduced by HFD and increased by APS supplement, including taurine and hypotaurine metabolism, acarbose and validamycin biosynthesis, isoquinoline alkaloid biosynthesis, streptomycin biosynthesis, tropane, piperidine and pyridine alkaloid biosynthesis, nicotinate and nicotinamide metabolism, polyketide sugar unit biosynthesis, and zeatin biosynthesis (**Figure 3C**). In addition, glutathione metabolism was increased by HFD and tended to be reduced by APS (*p* = 0.07), while purine metabolism was significant reduced by HFD and tended to be increased by APS (*p* = 0.07). Altogether, the metabolic function of bacteria that changed by HFD could be partially reversed by APS supplementation.

### APS Reverses Metabolomic Changes in HFD-Fed Mice

**Figure 4:**
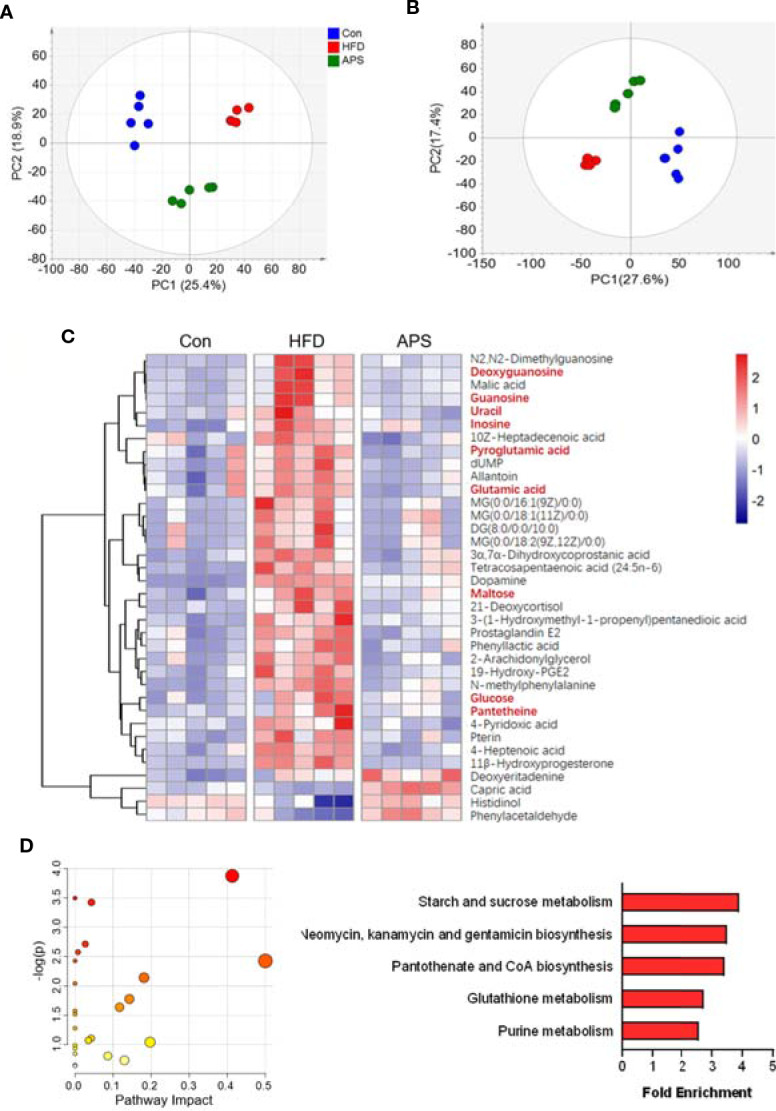
APS changes fecal metabolome in HFD-fed mice. The LC/MS-based untargeted metabolic profiling in positive and negative mode was performed on fecal samples (*n* = 5). The SIMCA positive-derived **(A)** and negative-derived **(B)** PCA among the Con, HFD, and APS groups. **(C)** Heatmap of differential metabolites identified in cecum samples between Con vs HFD, and HFD vs APS groups respectively with the double criteria of both VIP > 1 and *p* < 0.05. Each column represents an individual sample. **(D)** Bubble diagram of metabolic pathways enrichment based on the differential metabolites among three groups. One bubble represents one metabolic pathway. Top 5 pathways were listed on the right.

To elucidate the metabolic character of APS, the LC/MS-based untargeted metabolic profiling in positive and negative mode was performed on fecal samples. The unsupervised PCA, which was performed to visualize the general differences among samples, showed clear separation among groups in both positive and negative modes (**Figures 4A, B**). With the criteria of either VIP> 1 (multivariate statistical analysis) and *p* < 0.05 (univariate statistics), 36 metabolites were significantly altered by HFD and reversed by APS supplementation (**Figure 4C**). We next carried out the metabolic pathway analysis on the 36 differential metabolites. The top 5 significant altered pathways, which covered nine differential metabolites including deoxyguanosine, guanosine, uracil, inosine, pyroglutamic acid, glutamic acid, maltose, glucose, and pantetheine, were starch and sucrose metabolism, neomycin, kanamycin, and gentamicin biosynthesis, pantothenate and CoA biosynthesis, glutathione metabolism, and purine metabolism (**Figure 4D**). It is interesting to note that the glutathione metabolism and purine metabolism pathways were also identified based on altered bacterial function. These findings suggested that APS was effective in reversing HFD-induced dysregulated metabolism, which is associated with its effect on regulating gut microbiota composition.

### Correlation Between Gut Microbial Species and Differential Metabolites

**Figure 5:**
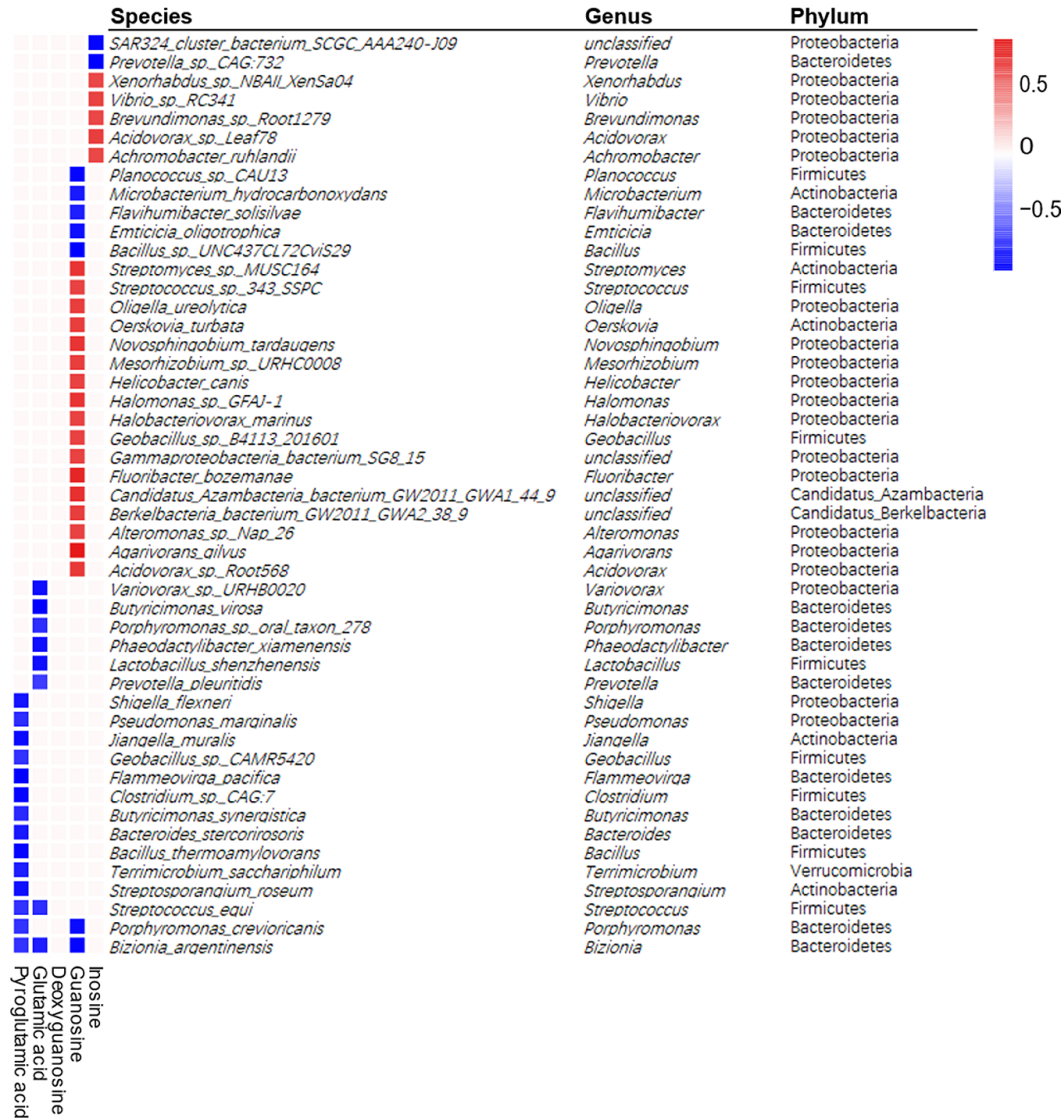
Spearman correlations between differential metabolites and differential bacterial species. Positive correlations indicated by red cubes and negative correlations indicated by blue cubes. A Spearman's correlation coefficient less than −0.7 or more than 0.7 with *p* < 0.01 was selected. The selected species were shown with its phylum and genus information.

Since two important metabolic pathways, glutathione metabolism and purine metabolism, were found both in metagenomic function analysis and metabolites pathway enrichment analysis, we investigated the correlation of 188 differential bacterial species with five differential metabolites enriched in these two pathways by spearman's correlation analysis. The heatmap revealed 49 species were correlated with four metabolites (**Figure 5**). The correlation analysis showed that the differential metabolites from glutathione metabolism, including pyroglutamic acid and glutamic acid, showed negative correlation with 14 and 8 differential bacterial species, respectively, with *Streptococcus_equi* and *Bizionia_argentinensis* negatively correlated with both metabolites. In addition, guanosine and inosine, which are from purine metabolism pathway, were positively correlated with 17 and 5 differential bacterial species respectively, and showed negative correlation with seven and two species respectively. Interestingly, deoxyguanosine from purine pathway was not correlated with any species. These results suggested certain bacteria that were shifted by APS were correlated with metabolites in glutathione metabolism and purine metabolism, indicating the important roles of these bacteria in APS-associated beneficial effects.

## Discussion

The beneficial effects of herbal polysaccharides on metabolic disorders have been shown to closely related to the alternation of gut microbiota composition and function as well as metabolites. In our current study, we performed metagenomic sequencing and untargeted metabolomics to uncover the regulatory mechanism of APS on host health. The metagenomic and metabolic profiling revealed that APS partially reversed HFD induced changes of bacterial structure and function as well as metabolism. Purine metabolism pathway and glutathione metabolism pathway might play important roles on the improvement of metabolic disorders by APS.

The gut microbiota composition has been shown to closely link with host health. Our data showed HFD increased the abundance of Firmicutes and decreased abundance of Bacteroidetes, while APS was effective in reversing these changes. A large number of clinical studies show that fecal microbiota from patients with non-alcoholic fatty liver and cirrhotic contained an increased abundance of Firmicutes and a decreased abundance of Bacteroidetes (Wei et al., 2016; Sookoian et al., 2020). Increased Firmicutes can produce more lipopolysaccharide and deoxycholic acid, which pass into the liver through hepatic portal vein, leading to inflammation in the liver (Yoshimoto et al., 2013; Bourzac, 2014). Bacteroidetes, that mostly inhabits the distal gut, participates in the fermentation of indigestible polysaccharides, such as dietary fiber includes cellulose, hemicellulose, β-glucan, to produce SCFAs (Salyers et al., 1977; Koropatkin et al., 2012). SCFAs can directly activate G protein-coupled-receptors (GPCRs), inhibit histone deacetylases, and serve as energy substrates, thus regulate various physiological processes and may contribute to health (Henao-Mejia et al., 2012; Bourzac, 2014). Interestingly, in our study, APS significantly reduced the abundance of Deferribacteres. Deferribacteres is a kind of bacteria that obtain energy through obligate or facultative anaerobic metabolism. Walker A et al., unraveled the nature and specificity of metabolic profiles related to gut ecology in obesity through combinatory approach using metabolomics and gut microbiome analysis, and found significant differences between the microbiome of the C57BL/6J mice (C57J, without obesity susceptibility) and the C57BL/6N mice (C57N, with obesity susceptibility) on phylum level of Deferribacteres, which propose an essential role of the microbiome in obesity susceptibility (Walker et al., 2014).

Polysaccharides can be used as carbon source for the growth of gut microbiota, which partially or completely fermented in the large intestine (El Kaoutari et al., 2013). Recent studies have noticed that polysaccharides intervention might be an efficient method to improve glycometabolism-related diseases by modulating specific bacteria associated with glycometabolism disorder (Jackson et al., 2018; Sanna et al., 2019). The polysaccharides from *Ophiopogon japonicus* (Thunb.) Ker Gawl. significantly improved the gut dysbiosis in obese mice, with the increase of the number of Lactobacillus (Shi et al., 2015). The intake of *Ganoderma lucidum* polysaccharides alleviated obesity on mice, with reduced Firmicutes to Bacteroidetes ratio in the intestinal tract (Chang et al., 2017), which is consistent with our finding.

The gut microbiota exerts an enormous impact on the health status of the host *via* modulation of its metabolic functions. In the present study, we found six bacterial pathways were increased and eight bacterial pathways were reduced by HFD based on metagenomic sequencing, and APS could reverse thus changes. For example, tryptophan metabolism and methane metabolism were significantly enriched by HFD and reversed by APS supplementation. Tryptophan metabolism has a central role in physiology and physiopathology. Disorder of tryptophan metabolism has been linked to irritable bowel syndrome, metabolic syndrome, obesity, infectious diseases, and neuropsychiatric disorders (Agus et al., 2018; Platten et al., 2019). In addition, methane metabolism is associated with obesity. Several studies suggested that methane-produced bacteria were significantly negatively associated with the percentage of visceral fat (Visconti et al., 2019). Moreover, nicotinate and nicotinamide metabolism pathway were reduced by HFD intake and increased by APS. In obese human subjects, low nicotinate intake is associated with reduced α-diversity and Bacteroidetes abundance in the microbiome (Fangmann et al., 2018). In humans *in vivo*, gut-targeted delayed-release nicotinate significantly increases Bacteroidetes, with an improvement of biomarkers for systemic insulin sensitivity and metabolic inflammation (Fangmann et al., 2018). Our current study suggested that differential shifted bacterial pathways that induced by HFD and reversed by APS can explain the beneficial effect of APS on improving metabolic disorders to a certain extent.

Gut metabolites, which are jointly generated by host and gut microbiota, play essential roles in maintaining host health. In order to find out the differential metabolites that involved in the beneficial effect of APS, we analyzed metabolite profile and metabolic pathway changes using cecum samples. The starch and sucrose metabolism pathway, which contains two differential metabolites glucose and maltose, showed the highest fold enrichment. A number of studies have shown that long-term HFD feeding result in dysregulated glucose homeostasis and insulin resistance (Luck et al., 2019), and our results confirmed that APS could normalize the fasting blood glucose and insulin levels in HFD-fed mice. In addition, purine metabolism and glutathione metabolism were reduced and increased by HFD, respectively, and tended to be reversed by ASP supplementation in metagenomic function analysis. Meanwhile, purine metabolism and glutathione metabolism were also significantly changed based on metabolites pathway enrichment analysis. Three differentially changed metabolites deoxyguanosine, guanosine, and inosine are from the purine metabolism pathway. The dysfunction of purine metabolism has drastic physiological and pathological consequences (Daignan-Fornier and Pinson, 2019). Additionally, purine metabolic pathway is involved in various inflammatory processes (Crittenden et al., 2018), and its end product, uric acid, is associated with a series of metabolic disorders, including insulin resistance, obesity, NAFLD, and chronic kidney disease (Ndrepepa, 2018). Moreover, we noticed that different species were correlated with these three metabolites with no overlap, suggesting that these differential species might involve in different stages of purine metabolism pathway. Two differential metabolites, pyroglutamic acid and glutamic acid, which were changed by HFD and reversed by APS, are included in glutathione metabolism pathway. Glutathione plays critical roles in protecting cells from oxidative damage and the toxicity of xenobiotic electrophiles and maintaining redox homeostasis (Wu et al., 2004). Glutamic acid and pyroglutamic acid are intermediates in the glutathione metabolism. Elevated glutamic acid and pyroglutamic acid levels are involved in impaired glutathione metabolism (Emmett, 2014; Liu et al., 2014). Our data suggested that APS reduced these two metabolites, which were increased by HFD intake, indicating the protective effect of APS on normalizing glutathione metabolism. In addition, we also found 20 differential species, including *Streptococcus_equi* and *Bizionia_argentinensis*, were negatively correlated with glutamic acid and pyroglutamic acid, suggesting these species might participate in the effects of APS on altering glutathione metabolism.

Altogether, our current study highlights that APS has beneficial effects on reversing HFD-induced metabolic disorders by regulating intestinal metabolism as well as gut microbial structure and function. The gut microbial alteration of APS mice is correlated with the changes of metabolites in the cecum. Additionally, purine metabolism and glutathione metabolism were found both in metagenomic function analysis and metabolites pathway enrichment analysis, suggesting the importance of these two pathways in APS-associated improvement of metabolic disorders. In conclusion, our current study provides novel evidence of the underlying mechanisms of APS in treating metabolic disorders at both gut microbiota and metabolism levels.

## Data Availability Statement

The datasets presented in this study can be found in online repositories. The names of the repository/repositories and accession number(s) can be found in the article/**Supplementary Material**.

## Ethics Statement

The animal study was reviewed and approved by the institutional Animal Ethics Committee, Shanghai University of Traditional Chinese Medicine.

## Author Contributions

YH and BL conducted the *in vivo* experiments, data analysis, and manuscript writing. NZ and GW analyzed LC/MS-based untargeted metabolomic. JM, XT, and LC helped in animal experiment. JZ helped in H&E staining of tissues and hepatic steatosis evaluation. LS helped in data analysis and manuscript revision. HL supervised the project and revised the manuscript.

## Funding

This work was funded by National Natural Science Foundation of China (No. 81873059 and 81673662), National Key Research and Development Program of China (No. 2017YFC1700200), and Program for Professor of Special Appointment (Eastern Scholar) and Shuguang Scholar (16SG36) at Shanghai Institutions of Higher Learning from Shanghai Municipal Education Commission.

## Conflict of Interest

The authors declare that the research was conducted in the absence of any commercial or financial relationships that could be construed as a potential conflict of interest.
